# Promoting hand hygiene in intensive care: “PCI Licence”

**DOI:** 10.1186/2047-2994-4-S1-P284

**Published:** 2015-06-16

**Authors:** D Joubert, C Landelle, E Genevois, J Pugin, S Harbarth, D Pittet

**Affiliations:** 1 Infection Control Program, HUG, Switzerland; 2 Intensive Care Unit (ICU), HUG, Switzerland

## Introduction

Improving hand hygiene (HH) compliance among healthcare workers (HCWs) in ICUs requires innovative, specific and customized strategies.

## Objectives

To implement an innovative training strategy to improve HH compliance and decrease MRSA attack rates in a large (36 beds) mixed adult ICU.

## Methods

We used an innovative multimodal approach (“PCI License”) for HH training, based on courses originally designed within the aviation regulation EASA and the risk management program “Air crew”. Efforts, focused on ICUs and HCWs, were built on theoretical as well as practical components and included a video, in which the participant him/herself is placed as a HH observer. Course completion required a final skill/knowledge examination requiring 75 % minimum to pass. We monitored HH according to the WHO “My Five Moments for Hand Hygiene” concept. The attack rate of methicillin-resistant *Staphylococcus aureus* (MRSA) cross-transmission was monitored based on active surveillance screening.

## Results

Between June 2013 and June 2014, HCWs of the Infection Control program and ICU conducted together 32 training courses. A total of 170/230 (74%) nurses and nursing assistants and 26/32 (81%) physicians followed the training. Skill/knowledge evaluation results were the following: success, 75% or more: 61 % (n = 140); partial success, between 75% and 50%: 11 % (n = 25); failure, lower than 50%: 5% (n = 11); 20 (10%) staff were reviewed individually. In 2014, 910 opportunities for HH were observed in the ICU; overall performance was 66.2% compared to 64.8% in 2013 (486 opportunities) and 60.4% in 2012 (840 opportunities). The MRSA attack rate decreased in parallel from 2.4 ICU-acquired cases/1000 patient-days in 2012 to 2.3 in 2013, and 1.3 in 2014.

## Conclusion

ICU staff compliance with HH improved following the proposed multimodal approach, while MRSA attack rates decreased. The program proved to be an excellent example of collaboration between ICU and infection control staff, working together to reduce the burden of ICU-acquired infections.

## Disclosure of interest

None declared.

